# A Young Man Evaluated for Suspicion of Lymphoma

**DOI:** 10.4269/ajtmh.14-0084

**Published:** 2014-09-03

**Authors:** Anthony P. Cannella, Joseph M. Vinetz

**Affiliations:** Division of Infectious Diseases, Department of Medicine, University of California, San Diego, California

**Figure 1.**
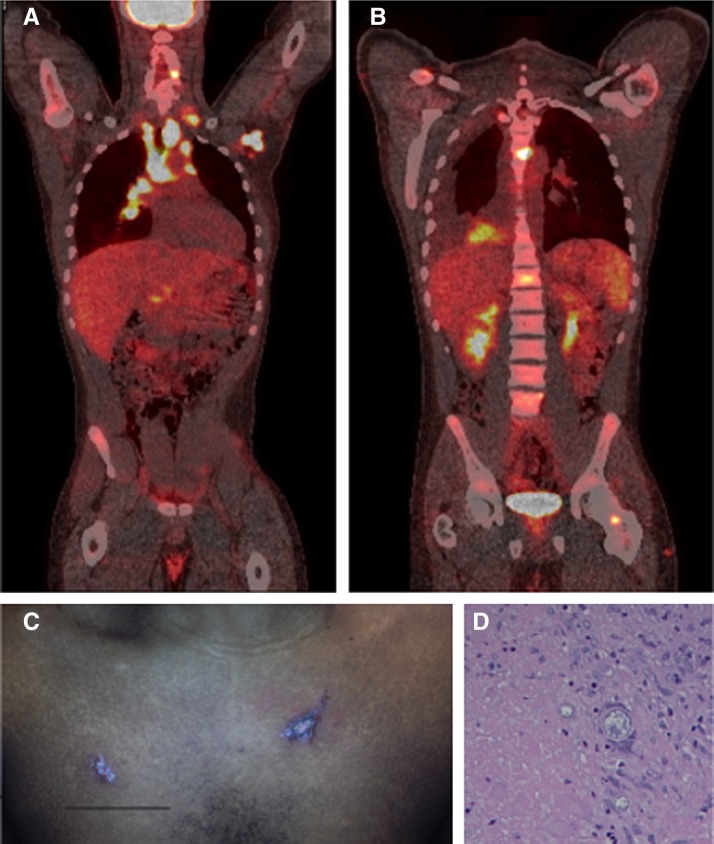
(**A** and **B**) Positron emission tomography. (**A**) Coronal section-anterior view; (**B**) coronal section-posterior view) of patient with coccidioidomycosis; noted tracer uptake in axillary and mediastinal lymph nodes, cervical and lumbar vertebrae, left ilium, and perineum. (**C**) Integumenal lesions on patient's anterior chest. (**D**) Hematoxylin and eosin (H&E) stain of left axillary lymph node biopsy showing *Coccidioides* spp. spherules within a granuloma.

A 21-year-old man had moved to Riverside County, California from Botswana at 7 years of age. He presented with B symptoms: intermittent fever, fatigue, and 20 kg weight loss progressive over 6 weeks. He had no relevant past medical history; his last trip to Africa was in 2002. On examination, he had axillary and inguinal lymphadenopathy; nodular skin ulcerations were present on the forehead, fingers, scalp, and chest (Figure 1C). Laboratory studies showed low hemoglobin (10.7 g/dL), a negative *Mycobacterium tuberculosis* interferon-γ release assay, and a negative serology for human immunodeficiency virus. A concern for lymphoma prompted a positron emission computer tomography scan (PET CT-coronal images shown), which showed extensive tracer uptake in mediastinal and axillary lymph nodes (Figure 1A-anterior coronal image shown), vertebral bodies and pedicles, and the right testis (Figure 1B-posterior coronal image shown).^1^ An excisional biopsy (hematoxylin and eosin [H&E] stain) of a left axillary lymph node showed spherules consistent with *Coccidioides* species (Figure 1D). The serum *Coccidioides* complement fixation titer was 1:512; cerebrospinal fluid analysis was unremarkable. Fluconazole 800 mg/d by mouth led to clinical resolution. Infections with *Coccidioides immitis/posadasii* are common in the southwestern United States, Mexico, and parts of South America,^2^ and has recently been found to be expanding in range to previously unsuspected areas such as Washington State.^3^
